# Surgical management of carcinoma in situ at ductal resection margins in patients with extrahepatic cholangiocarcinoma

**DOI:** 10.1002/ags3.12196

**Published:** 2018-07-26

**Authors:** Toshifumi Wakai, Jun Sakata, Tomohiro Katada, Yuki Hirose, Daiki Soma, Pankaj Prasoon, Kohei Miura, Takashi Kobayashi

**Affiliations:** ^1^ Division of Digestive and General Surgery Niigata University Graduate School of Medical and Dental Sciences Niigata Japan

**Keywords:** additional resection, bile duct neoplasm, carcinoma in situ, cholangiocarcinoma, ductal resection margin status

## Abstract

Recent advances in dimensional imaging, surgical technique, and perioperative patient care have resulted in increased rates of complete resection with histopathologically negative margins and improved surgical outcomes in patients with extrahepatic cholangiocarcinoma. However, achieving cancer‐free resection margins at ductal stumps in surgery for this disease remains challenging because of longitudinal extension, which is one of the hallmarks of extrahepatic cholangiocarcinoma. When the ductal resection margins are shown to be positive on examination of frozen sections, discrimination between carcinoma in situ and invasive carcinoma is clinically important because residual carcinoma in situ may lead to late local recurrence whereas residual invasive carcinoma is associated with early local recurrence. Residual invasive carcinoma at the ductal margins should be avoided whenever technically feasible. Residual “carcinoma in situ” at the ductal margins appears to be allowed in resection for the advanced disease because it has less effect on survival than other adverse prognostic factors (pN1 and/ or pM1). However, in surgery for early‐stage (pTis‐2N0M0) extrahepatic cholangiocarcinoma, residual carcinoma in situ at the ductal margins may have an adverse effect on long‐term survival, so should be avoided whenever possible. In this review, we focus on the histopathological term “carcinoma in situ,” the biological behavior of residual carcinoma in situ at ductal resection margins, intraoperative histological examination of the ductal resection margins, outcome of additional resection for positive ductal margins, and adjuvant therapy for patients with positive margins.

## 1 INTRODUCTION

Surgical resection with curative intent provides the best chance of cure and long‐term survival in patients with resectable extrahepatic cholangiocarcinoma.^1^, ^2^, ^3^, ^4^, ^5^, ^6^, ^7^, ^8^, ^9^, ^10^ Recent advances in dimensional imaging, perioperative management, including biliary drainage and percutaneous transhepatic portal vein embolization, and surgical technique have resulted in increased rates of complete resection with histopathologically negative margins and improved patient survival in patients with extrahepatic cholangiocarcinoma.^3^, ^4^, ^5^, ^6^ However, cancer‐free resection margins at the bile duct stump are difficult to achieve because of longitudinal extension, which is one of the prominent characteristics of extrahepatic cholangiocarcinoma.^11^, ^12^ Ductal resection margin status is an established prognostic indicator,^13^, ^14^ and survival following resection in patients with positive ductal margins has generally been deemed unsatisfactory.^10^, ^15^, ^16^, ^17^, ^18^, ^19^, ^20^, ^21^, ^22^, ^23^, ^24^, ^25^ In 2005, Wakai et al^26^ reported that invasive carcinoma at the ductal resection margins had a strong adverse effect on survival in patients with extrahepatic cholangiocarcinoma, whereas residual carcinoma in situ did not. Thereafter, similar results were reported in Japan,^27^, ^28^, ^29^, ^30^, ^31^, ^32^, ^33^, ^34^, ^35^, ^36^ the USA,^37^ South Korea,^38^, ^39^ and Germany.^40^ These findings indicate that discrimination between carcinoma in situ and invasive carcinoma is critical when the ductal resection margins are found to be positive on intraoperative examination of frozen sections; residual carcinoma in situ may lead to late local recurrence, whereas residual invasive carcinoma results in early local recurrence.^26^

Herein, we review the surgical management of carcinoma in situ at the ductal resection margins in patients undergoing curative‐intent resection for extrahepatic cholangiocarcinoma, including perihilar cholangiocarcinoma and distal bile duct cancer. This review focuses on the histopathological term “carcinoma in situ,” its biological behavior at the ductal resection margins, intraoperative histological examination of these margins, outcome of additional resection for positive ductal margins in perihilar cholangiocarcinoma, and adjuvant therapy for patients with positive ductal margins.

## 2 “CARCINOMA IN SITU”: HISTOPATHOLOGICAL TERMINOLOGY

In the WHO International Histological Classification of Tumours: Histological Typing of Tumours of the Gallbladder and Extrahepatic Bile Ducts published in 1991, Albores‐Saavedra et al^41^ discussed dysplasia and carcinoma in situ together because of the problems in distinguishing between these two entities. Dysplasia is defined as epithelial atypia, in which the risk of progression to carcinoma is higher than that in normal epithelium.^41^ Dysplasia is histologically characterized by columnar, cuboidal, or elongated cells that show varying degrees of pseudostratification, nuclear atypia, loss of polarity, and mitotic figures.^41^ Carcinoma in situ is epithelium that has the histological characteristics of carcinoma but no evidence of invasion to the lamina propria.^41^ It is believed that dysplasia‐carcinoma in situ is the usual sequence for development of carcinoma of the extrahepatic biliary tract; however, a small number of carcinomas develop from preexisting adenomas. Differentiation of dysplasia or carcinoma in situ from regenerative epithelial atypia may be difficult. This distinction is of clinical significance because regenerative epithelial atypia is not precancerous.

In Tumors of the Gallbladder, Extrahepatic Bile Ducts, and Ampulla of Vater, published by Armed Forces Institute of Pathology (AFIP) in 2000, Albores‐Saavedra et al^42^ described the foci of dysplasia and carcinoma in situ as being multicentric in most cases, a finding that probably has therapeutic implications and explains the high incidence of local recurrence. Differentiation between severe dysplasia and carcinoma in situ may be difficult or impossible. Therefore, in several clinical studies,^26^, ^27^, ^28^, ^35^, ^36^, ^38^, ^39^ severe (high‐grade) dysplasia has been categorized as carcinoma in situ. Severe dysplastic epithelium or carcinoma in situ may extend into intramural glands such as the sacculi of Beale or metaplastic pyloric type glands.^42^ Distinction between these intramural epithelial lesions (pseudoinvasion) and invasive carcinoma is now made according to the histological criteria defined by Albores‐Saavedra et al.^42^

The term “intraepithelial neoplasia” (encompassing dysplasia and carcinoma in situ) was initially used in the WHO Classification of Tumours of the Digestive System^43^ in 2000. The differentiation between high‐grade intraepithelial neoplasia (severe dysplasia) and carcinoma in situ is difficult and may be impossible in many cases.^43^ In the 2010 WHO Classification of Tumours of the Digestive System,^44^ the term “biliary intraepithelial neoplasia, grade 3” (BilIN‐3) was implemented. BilIN often arises in association with chronic cholecystolithiasis and is not usually detected on macroscopic examination; it is typically detected incidentally and is of no established clinical significance.^44^ When associated with an invasive carcinoma, the morphological type of BilIN‐3 does not always correspond with that of the carcinoma. BilIN‐3 includes so‐called “carcinoma in situ”.^45^

In Tumors of the Gallbladder, Extrahepatic Bile Ducts, and Vaterian System, published by American Registry of Pathology, AFIP, in 2015, Albores‐Saavedra et al^46^ reported that separation of high‐grade dysplasia from carcinoma in situ in the extrahepatic bile ducts is subjective and often not possible. This distinction is further complicated by the lack of established morphological criteria for high‐grade dysplasia/carcinoma in situ. Therefore, these two lesions should be included in a single group; that is, high‐grade dysplasia/carcinoma in situ. The dysplasia‐carcinoma sequence is the usual pathway for progression to invasive carcinoma from the extrahepatic bile ducts.^47^, ^48^, ^49^, ^50^, ^51^, ^52^ Non‐invasive papillary carcinomas do not metastasize, and complete excision may be curative, so extensive sampling is recommended to exclude invasion.^47^

According to the 8th edition of the AJCC Cancer Staging Manual^2^ published in 2017, Tis is defined as carcinoma in situ/high‐grade dysplasia. The definition of Tis has been expanded to include high‐grade biliary intraepithelial neoplasia (BilIN‐3), which is a non‐invasive neoplastic process, that is synonymous with carcinoma in situ.^2^ Tumors classified as Tis cytologically resemble carcinoma, with diffuse and severe distortion of cellular polarity, but invasion through the basement membrane is absent.

## 3 BIOLOGICAL BEHAVIOR OF RESIDUAL CARCINOMA IN SITU

Reported incidences of residual carcinoma in situ and invasive carcinoma at the ductal resection margins in patients who have undergone resection of extrahepatic cholangiocarcinoma have been in the range of 3%‐16% and 8%‐18.3%, respectively (Table 1).^26^, ^27^, ^28^, ^31^, ^32^, ^34^, ^35^, ^38^ The reported incidences of complete resection with histopathologically negative margins ranged from 69% to 87%.^26^, ^27^, ^28^, ^31^, ^32^, ^34^, ^35^, ^38^ All the studies included in Table 1 confirmed that ductal resection margin status was an independent prognostic factor in patients with extrahepatic cholangiocarcinoma. Residual invasive carcinoma at the ductal resection margins (median survival time, 12‐21 months) has been reported to influence patient survival after surgical resection for extrahepatic cholangiocarcinoma more adversely than residual carcinoma in situ (median survival time, 29‐99 months).^26^, ^27^, ^28^, ^31^, ^32^, ^34^, ^38^ Several research groups reported that survival following resection was comparable between patients who had negative ductal margins (median survival time, 33‐55 months) and those who had positive ductal margins with carcinoma in situ (median survival time, 37‐99 months).^26^, ^27^, ^28^, ^31^, ^32^, ^35^ Although residual carcinoma in situ at the ductal margins does not have a strong adverse effect on survival in patients with extrahepatic cholangiocarcinoma, it may result in late local recurrence.^26^, ^27^, ^28^, ^29^, ^31^, ^32^, ^34^, ^36^, ^53^, ^54^, ^55^, ^56^, ^57^

**Table 1:** Impact of residual carcinoma in situ at ductal resection margins on surgical outcomes in patients with extrahepatic cholangiocarcinoma

No.	Author	Year	Location	Ductal resection margin status	No. of patients (%)	5‐y survival rate (%)	MST (mo)	Comparison^a^	*P*‐value
1	Wakai et al^26^	2005	Perihilar, n = 41; Distal, n = 43	R0	64 (76)	46	45	R1 CIS vs R0	0.4742
				R1 CIS	11 (13)	69	99	R1 CIS vs **R1 invasive**	0.0003
				R1 invasive	9 (11)	0	21		
2	Sasaki et al^27^	2007	Perihilar, n = 51; Distal, n = 77	R0	105 (82)	35.5	33	R1 CIS vs R0	0.5247
				R1 CIS	12 (9.4)	22.2	37	R1 CIS vs **R1 invasive**	0.0241
				R1 invasive	11 (8.6)	0	12		
3	Igami et al^28^	2009	Perihilar, n = 351; Distal, n = 120	R0	410 (87)	32.0	ND	R1 CIS vs R0	0.398
				R1 CIS	14 (3)	0	ND	R1 CIS vs **R1 invasive**	0.015
				R1 invasive	47 (10)	10.8	ND		
4	Nakanishi et al^31^	2010	Perihilar, n = 103; Distal, n = 22	R0	96 (77)	32	38	R1 CIS vs R0	0.533
				R1 CIS	10 (8)	48	51	R1 CIS vs **R1 invasive**	0.006
				R1 invasive	19 (15)	NE	17		
5	Higuchi et al^32^	2010	Perihilar, n = 80; Distal, n = 135	R0	185 (86)	54.7	ND	R1 CIS vs R0	NS
				R1 CIS	13 (6)	52.4	ND	R1 CIS vs **R1 invasive**	0.0030
				R1 invasive	17 (8)	17.6	ND		
6	Han et al^38^	2014	Perihilar, n = 208; Distal, n = 246; Diffuse, n = 10	R0	340 (73.3)	44.5	41	**R1 CIS** vs R0	<0.001
				R1 CIS	39 (8.4)	20.7	29	R1 CIS vs **R1 invasive**	0.029
				R1 invasive	85 (18.3)	12.0	18		
7	Tsukahara et al^34^	2017	Perihilar, n = 144; Distal, n = 28 (pTis‐T2N0M0)	R0	148 (86)	78.7	NE	**R1 CIS** vs R0	0.005
				R1 CIS	18 (10.5)	35.1	53	R1 CIS vs **R1 invasive**	0.002
				R1 invasive	6 (3.5)	NE	13		
8	Kurahara et al^35^	2017	Perihilar, n = 35; Distal, n = 65	R0	69 (69)	ND	55	R1 CIS vs R0	0.240
				R1 CIS	16 (16)	ND	53	R1 CIS vs R1 invasive	0.418
				R1 invasive	15 (15)	ND	24		

a Patient group showing significantly unfavorable outcomes when compared with the other groups are shown in bold underlined text.

R0, a negative ductal resection margin; R1 CIS, a positive ductal resection margin with carcinoma in situ; R1 invasive, a positive ductal resection margin with invasive carcinoma; MST, median survival time; ND, not described; NE, not evaluated; NS, not statistically significant.

Some authors have reported a statistically significant association of status of the ductal resection margins with local recurrence.^27^, ^31^, ^32^ However, the likelihood of recurrence depends on the duration of follow up. Using the Cox proportional hazards regression model, Wakai et al^58^ demonstrated that the ductal resection margin status was the only factor that was independently associated with local recurrence in patients with residual carcinoma in situ and invasive carcinoma, with a relative risk for local recurrence of 4.26 and 7.00, respectively.

In 2009, Ojima et al^29^ reported adjusted hazard ratios and 95% confidence intervals for survival in patients with residual carcinoma in situ and residual invasive carcinoma at the ductal resection margins of 1.06 (0.53‐2.10) and 1.95 (1.27‐3.00), respectively. They proposed that surgeons do not need to persist in their attempts to achieve negative ductal resection margins when a diagnosis of residual carcinoma in situ is made on intraoperative examination of frozen sections.^29^ In 2011, Wakai et al^58^ reported that after stratification based on pN and pM classification, the ductal resection margin status in patients with extrahepatic cholangiocarcinoma significantly influenced long‐term survival following resection in those with pN0pM0 disease but not in those with pN1 and/or pM1 disease. When the ductal resection margin status is shown to be carcinoma‐positive on examination of frozen sections, additional resection should be considered in patients with localized (pN0pM0) disease.^28^, ^34^, ^36^, ^47^, ^58^, ^59^, ^60^ In 2017, Tsukahara et al^34^ first reported that residual carcinoma in situ at the bile duct stumps increased the incidence of local recurrence and adversely affected postoperative survival in patients who underwent resection for early‐stage (pTis‐2N0M0) cholangiocarcinoma.

## 4 INTRAOPERATIVE HISTOLOGICAL EXAMINATION OF DUCTAL RESECTION MARGINS

Ductal resection margin status in patients with extrahepatic cholangiocarcinoma has traditionally been evaluated intraoperatively by histological examination of frozen sections.^26^, ^27^, ^29^, ^33^, ^37^, ^61^, ^62^ In 2009, Konishi et al^30^ proposed a new histological classification of ductal resection margins on intraoperative frozen‐section examination in cholangiocarcinoma. However, distinction between severe (high‐grade) dysplasia, carcinoma in situ, and intraepithelial neoplasia is subjective.^46^ The distinction is further complicated by the lack of established morphological criteria for intraepithelial lesions including severe (high‐grade) dysplasia, carcinoma in situ, and intraepithelial neoplasia.^46^ Severe dysplastic epithelium or carcinoma in situ may extend into intramural glands, such as the sacculi of Beale and metaplastic pyloric‐type glands.^26^, ^41^, ^42^, ^43^ Such intramural epithelial lesions (pseudoinvasion) are distinguished from invasive carcinoma according to the histological criteria defined by Albores‐Saavedra et al^42^

## 5 OUTCOME OF ADDITIONAL RESECTION FOR CARCINOMA‐POSITIVE DUCTAL MARGINS

In clinical practice, additional intraoperative resection of the proximal bile duct is often carried out for perihilar cholangiocarcinoma to obtain a negative ductal margin based on examination of frozen sections. However, the impact of this practice on the surgical outcomes of perihilar cholangiocarcinoma remains controversial (Table 2). In 2008, Endo et al^37^ reported on the clinical significance of proximal ductal resection margins in 101 patients with perihilar cholangiocarcinoma. They divided the proximal ductal resection margin status of these patients into three categories based on final pathological examination as follows: a wide margin (both an additional ductal resection margin and specimen margin negative, n = 54), a narrow margin (an additional ductal resection margin negative but specimen margin positive, n = 28), and a positive margin (both an additional ductal resection margin and specimen margin positive, n = 19).^37^ Survival in patients with a narrow margin was significantly worse than that in patients with a wide margin and was comparable with that in patients with a positive margin (Table 2).^37^ The results of their study suggested that surgical outcome is not altered by extending the resection of the proximal bile duct in most patients with perihilar cholangiocarcinoma.

**Table 2:** Impact of additional resection for positive ductal resection margins on surgical outcomes in patients with perihilar cholangiocarcinoma

No.	Author	Year	Proximal ductal resection margin status^a^	No. of patients (%)	5‐y survival rate (%)	MST (mo)	Comparison^b^	*P*‐value
1	Endo et al^37^	2008	Primary R0	54 (53)	43	56	**Secondary R0 and R1** vs Primary R0	0.010
			Secondary R0	28 (28)	18	38		
			R1	19 (19)	ND	32		
2	Shingu et al^57^	2010	Primary R0	275 (90.8)	37	ND	**Secondary R0** vs Primary R0	0.022
			Secondary R0	8 (2.6)	0	ND	Secondary R0 vs R1	0.294
			R1	20 (6.6)	16	ND		
3	Ribero et al^63^	2011	Primary R0	54 (72)	30.8	29.2	Secondary R0 vs Primary R0	NS
			Secondary R0	13 (17)	50	30.6	Secondary R0 vs **R1**	0.026
			R1	8 (11)	0	14.9		
4	Oguro et al^64^	2015	Primary R0	149 (67)	48.6	56.6	**Secondary R0** vs Primary R0	0.031
			Secondary R0	43 (19)	30	29.4	Secondary R0 vs R1^c^	0.215
			R1	32 (14)	16.8	21.5		
5	Zhang et al^65^, ^d^	2018	Primary R0	136 (53)	23.3	22.3	Secondary R0 vs Primary R0	0.804
			Secondary R0	29 (11)	44.3	30.6	Primary R0 vs R1	0.088
			R1	92 (36)	7.9	18.5		

a Positive ductal resection margins with carcinoma in situ were treated as negative ductal resection margins.

b Patient groups with significantly unfavorable outcomes compared with the other groups are shown in bold and underlined text.

c Patients with secondary R0 had significantly better outcomes than those with R1 only if they had a lower CA19‐9 level and no distant metastatic disease.

d In this study, both proximal and distal ductal resection margin status were evaluated.

Primary R0, a negative ductal resection margin without additional resection; Secondary R0, a negative ductal resection margin with additional resection; R1, a microscopic positive ductal resection margin; MST, median survival time; ND, not described; NS, not statistically significant.

Shingu et al^57^ reported the clinical importance of additional resection for positive proximal bile duct margins in 303 patients with perihilar cholangiocarcinoma, 12 of whom underwent additional resection after invasive carcinoma at the ductal margins was confirmed by frozen section examination. In all 12 patients, the length of the additional resection was ≤5 mm and a negative ductal margin was obtained by additional resection in 8 patients.^57^ Their results indicated that such limited resection (≤5 mm) for a positive proximal ductal margin was not associated with improved survival, even when a negative ductal margin was obtained by additional resection (Table 2). They proposed one possible reason for their results. All 8 patients with a negative proximal ductal margin after additional resection had at least one independent prognostic factor that contributed strongly to worse survival; for such patients, clearance of the proximal ductal margin might not confer any survival benefit because the status of the ductal margins has a less powerful influence on the outcome.^57^

In contrast, Ribero et al^63^ reported that additional resection of a positive proximal ductal margin offered a survival benefit in patients with perihilar cholangiocarcinoma. In their study, survival in patients with a negative proximal ductal margin achieved by additional resection was comparable with that in patients with a primary negative proximal margin and was significantly better than that in patients with a positive ductal margin (Table 2). They recommended that additional resection should be attempted for a positive proximal ductal margin whenever possible.^63^

Oguro et al^64^ attempted to clarify the optimal indications for additional resection of a positive proximal ductal margin in 224 patients with perihilar cholangiocarcinoma. Additional resection of a positive proximal ductal margin afforded no survival benefit in this study (Table 2). However, they demonstrated that, in the subgroups with a CA 19‐9 level <64 U/mL and pM0 disease, survival in patients with a negative proximal ductal margin who underwent additional resection was significantly better than that in patients with a positive proximal ductal margin. In addition, they attributed the inconsistent results between the different studies of the effect of additional resection of a positive proximal ductal margin on outcomes to differences in the tumor characteristics of the study population. The rates of Bismuth type IV disease in the studies by Oguro et al^64^ and Shingu et al^57^ were reported to be 40% and 38.9%, respectively, whereas the rate was 14.6% in the study by Ribero et al^63^ This suggests that the study by Ribero et al^63^ included more patients with less advanced tumors, where additional resection of a positive proximal ductal margin may have contributed to more favorable outcomes.

A study by Zhang et al^65^ that incorporated 10 high‐volume hepatobiliary centers throughout the USA investigated the impact of additional resection for a positive ductal margin on surgical outcomes in 257 patients with perihilar cholangiocarcinoma. Just like the results as reported by Ribero et al^63^ for their European center, Zhang et al^65^ demonstrated that additional resection of a positive proximal or distal ductal margin was associated with improved survival after curative‐intent resection in patients with perihilar cholangiocarcinoma (Table 2). They concluded that every attempt should be made to achieve a carcinoma‐negative ductal margin when technically feasible. In their study,^65^ the rate of Bismuth type IV disease was 22.6%, which was lower than the rates in the Eastern centers reported by Oguro et al^64^ and Shingu et al.^57^

Recently, Tsukahara et al^34^ reported the clinical importance of additional resection for a carcinoma in situ‐positive ductal margin in patients with early‐stage (Tis‐T2N0M0) cholangiocarcinoma. In their study, 12 patients underwent additional resection for carcinoma in situ at a ductal resection margin and a negative margin was achieved after additional resection in 7 patients. Survival in these 7 patients was comparable with that in patients with a primary negative margin for both invasive carcinoma and carcinoma in situ and was significantly better than that in patients with a carcinoma in situ‐positive ductal margin. The findings of that study suggest that additional resection for carcinoma in situ at the ductal resection margins confers a survival benefit in patients with early‐stage cholangiocarcinoma, with the caveat that a limited number of patients were investigated. Further studies are warranted to confirm the efficacy of this practice.

## 6 ADJUVANT THERAPY

Although surgery affords the only chance of cure in patients with extrahepatic cholangiocarcinoma, the surgical outcomes remain poor because of a high rate of recurrence. Despite curative‐intent resection, positive ductal resection margins are sometimes confirmed after pathological examination. Adjuvant therapy has been advocated to improve these poor outcomes.^66^, ^67^, ^68^, ^69^, ^70^, ^71^ However, given the rarity of extrahepatic cholangiocarcinoma, most of the data regarding adjuvant therapy have come from small, single‐center studies or retrospective single‐arm reviews. Recent relatively large studies and systematic reviews/meta‐analyses have suggested that adjuvant chemoradiotherapy, radiotherapy, or chemotherapy are associated with improved survival in patients with biliary tract cancer and high‐risk characteristics, including positive ductal resection margins.^66^, ^67^, ^68^ However, the intention‐to‐treat analyses in all the large randomized clinical studies of adjuvant chemotherapy for biliary tract cancer reported thus far have failed to demonstrate its efficacy.^69^, ^70^, ^71^ Therefore, the effective adjuvant chemotherapy regimen for this disease entity remains undetermined. Further clinical trials of adjuvant treatment focusing on patients with high‐risk characteristics are needed to resolve this problem. In the meantime, adjuvant therapy should be considered as a multimodal treatment option for patients with extrahepatic cholangiocarcinoma in whom positive ductal resection margins are confirmed after surgical resection.

## 7 CONCLUSIONS

Clinically, discrimination between carcinoma in situ and invasive carcinoma is essential when the ductal resection margins are found to be positive on examination of frozen sections in patients with extrahepatic cholangiocarcinoma. Patients with residual carcinoma in situ at the ductal resection margins may have late local recurrence, whereas residual invasive ductal lesions cause early local recurrence. Our recommendation for treatment of distinctive ailments of ductal resection margins in patients considering resection for extrahepatic cholangiocarcinoma is as follows. Residual carcinoma in situ at the ductal resection margins appears to be allowed in surgery for late‐stage disease because the status of these margins has less effect on survival than other adverse prognostic factors in this situation. Conversely, in surgery for early‐stage (pTis‐2N0M0) extrahepatic cholangiocarcinoma, residual carcinoma in situ at the ductal resection margins may have an adverse effect on long‐term survival, suggesting that residual carcinoma in situ at these margins should be avoided if possible in these patients. Residual invasive carcinoma at the ductal resection margins should also be avoided whenever technically feasible.

## DISCLOSURE

Conflicts of Interest: Authors declare no conflicts of interest for this article.
